# Supplementary material for: Histone H3K18 & H3K23 acetylation directs establishment of MLL-mediated H3K4 methylation
Source: J Biol Chem. 2024 Jul 1;300(8):107527. doi: 10.1016/j.jbc.2024.107527 (PMC11338103; doi:10.1016/j.jbc.2024.107527)
Supplement: Supporting Information [file mmc2.pdf]

Supporting Information for

Histone H3K18 & H3K23 acetylation directs establishment of MLL-mediated H3K4 methylation

Geoffrey C. Fox[1], Karl F. Poncha[2], B. Rutledge Smith[3], Lara N. van der Maas[3], Nathaniel N. Robbins[4], Bria Graham[4], Jill M. Dowen[3,5,6,7], Brian D. Strahl[1,3,7]*, Nicolas L. Young[2]*, and Kanishk Jain[3,7]*

[1] Curriculum in Genetics and Molecular Biology, University of North Carolina at Chapel Hill, School of Medicine, Chapel Hill, NC;

[2]Verna & Marrs McLean Department of Biochemistry and Molecular Pharmacology, Baylor College of Medicine, Houston, TX;

[3]Department of Biochemistry and Biophysics, University of North Carolina at Chapel Hill, School of Medicine, Chapel Hill, NC;

[4]EpiCypher, Inc., Durham, NC;

[5]Department of Biology, University of North Carolina at Chapel Hill, Chapel Hill, NC;

[6]Integrative Program for Biological and Genome Sciences, University of North Carolina at Chapel Hill, Chapel Hill, NC;

[7]Lineberger Comprehensive Cancer Center, University of North Carolina at Chapel Hill, School of Medicine, Chapel Hill, NC

*Co-corresponding authors: brian_strahl@med.unc.edu, nicolas.young@bcm.edu, and kanishk@med.unc.edu

Supplementary Figures S1-S4

Figure S1. Quantification of total H3 N-terminal tail acetylation.

Figure S2. Quantification of global methylation levels with and without HDAC inhibition.

Figure S3. Quantification of total H3 N-terminal tail methylation.

Figure S4. Determination of optimal enzyme concentrations for *in vitro* methylation and kinase assays.

Supplementary Table S1 (provided in separate Excel file)

Table S1. Middle-down MS data shown in main and supporting figures.

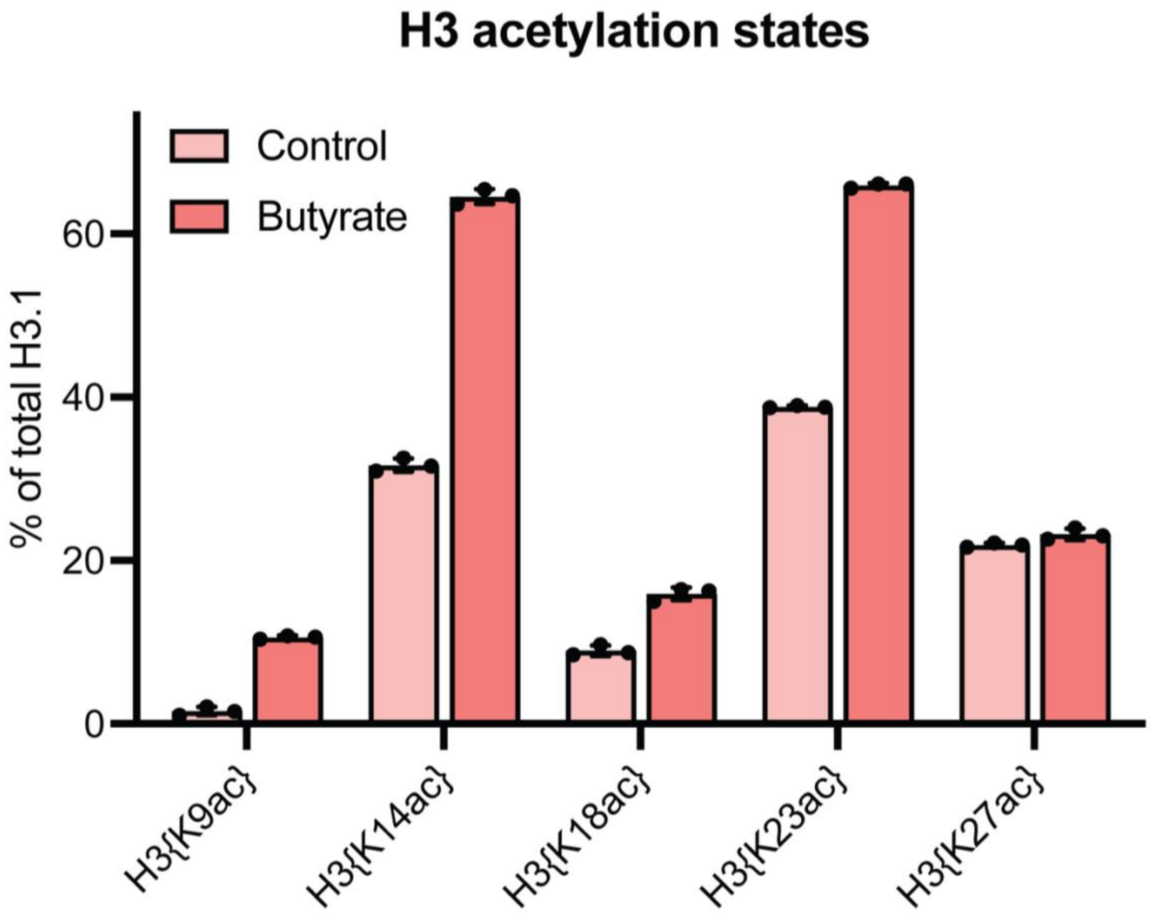

Supplementary Figure S1. Quantification of total H3 N-terminal tail acetylation. Acetylation states with (dark bars) and without (light bars) HDACi (butyrate) were quantified by middle-down mass spectrometry in HEK293 cells. MS data shown is reported in Supplementary Table 1. Data points shown are biological replicates. *n* = 3. Error: SD.

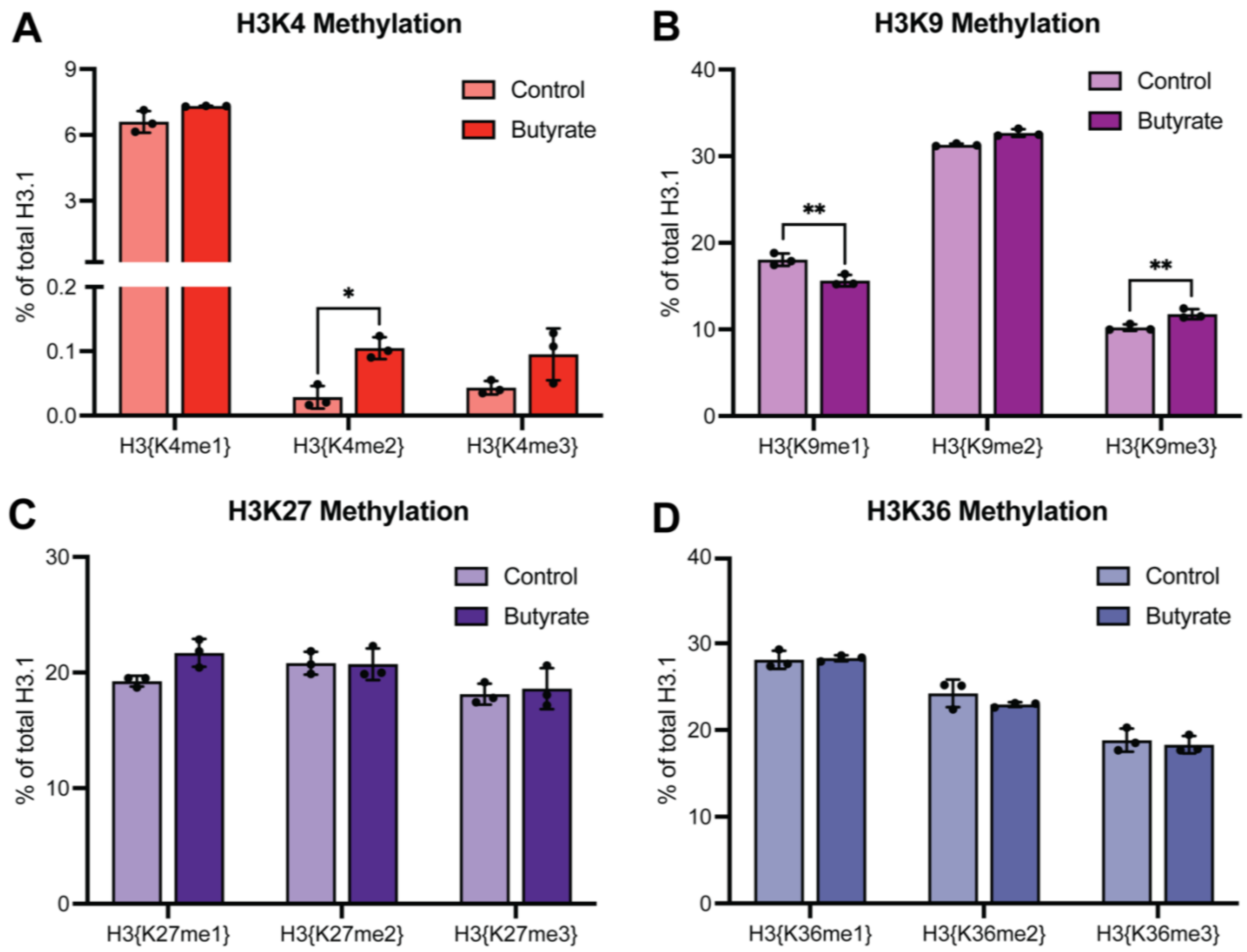

Supplementary Figure S2. Quantification of global methylation levels with and without HDAC inhibition. H3K4 (A), H3K9 (B), H3K27 (C), & H3K36 (D) methylation states with (dark bars) and without (light bars) HDACi (butyrate) were quantified by middle-down mass spectrometry in HEK293 cells. MS data shown is reported in Supplementary Table 1. Significance was determined by unpaired Student's t-test. NS unless otherwise designated. $*p < 0.05$, $**p < 0.005$. Data points shown are biological replicates. $n = 3$. Error: SD.

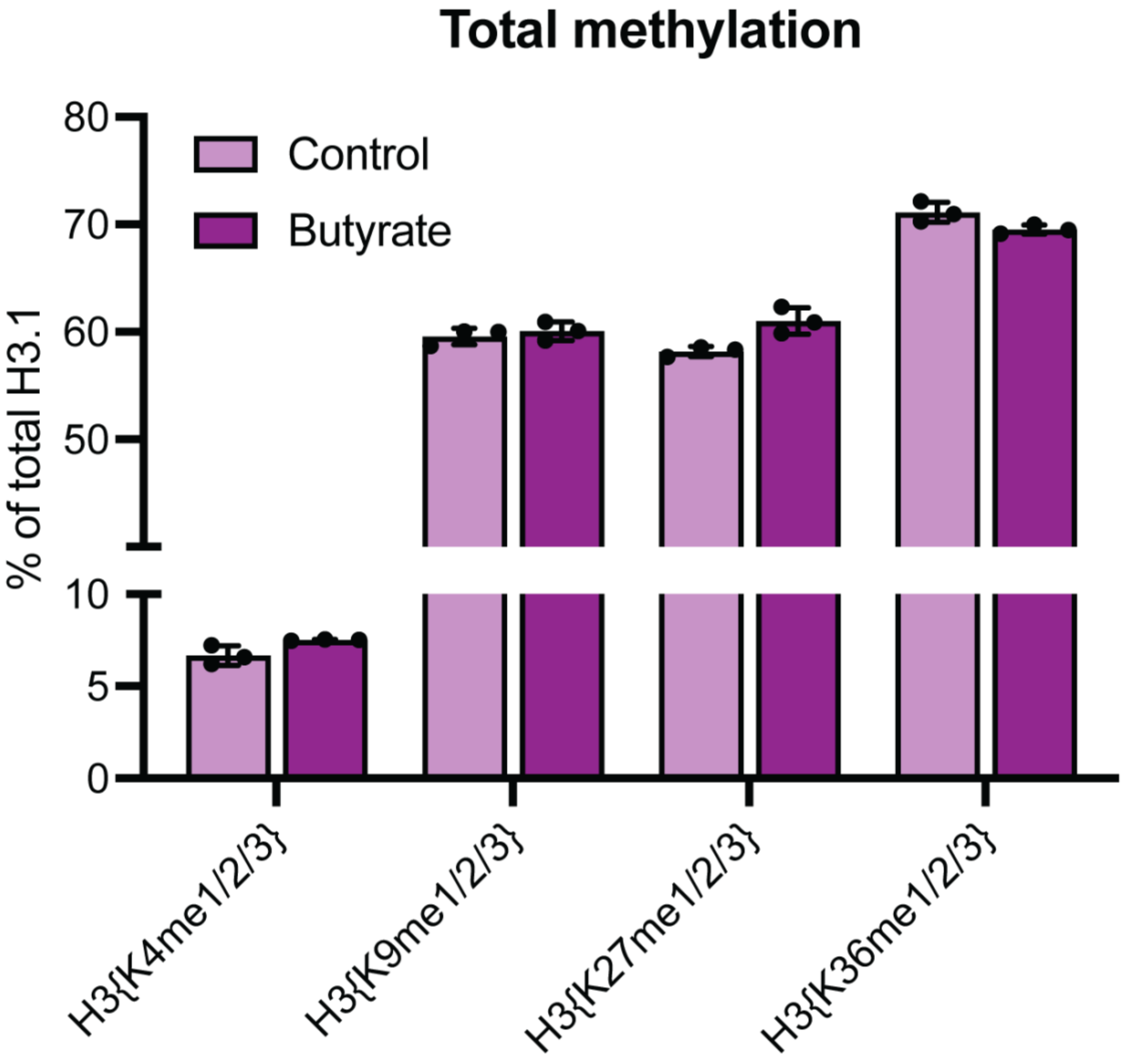

Supplementary Figure S3. Quantification of total H3 N-terminal tail methylation. Total methylation with (dark bars) and without (light bars) HDACi (butyrate) was quantified by middle-down mass spectrometry in HEK293 cells. MS data shown is reported in Supplementary Table 1. Significance was determined by unpaired Student's t-test. NS unless otherwise designated. Data points shown are biological replicates. Error: SD.

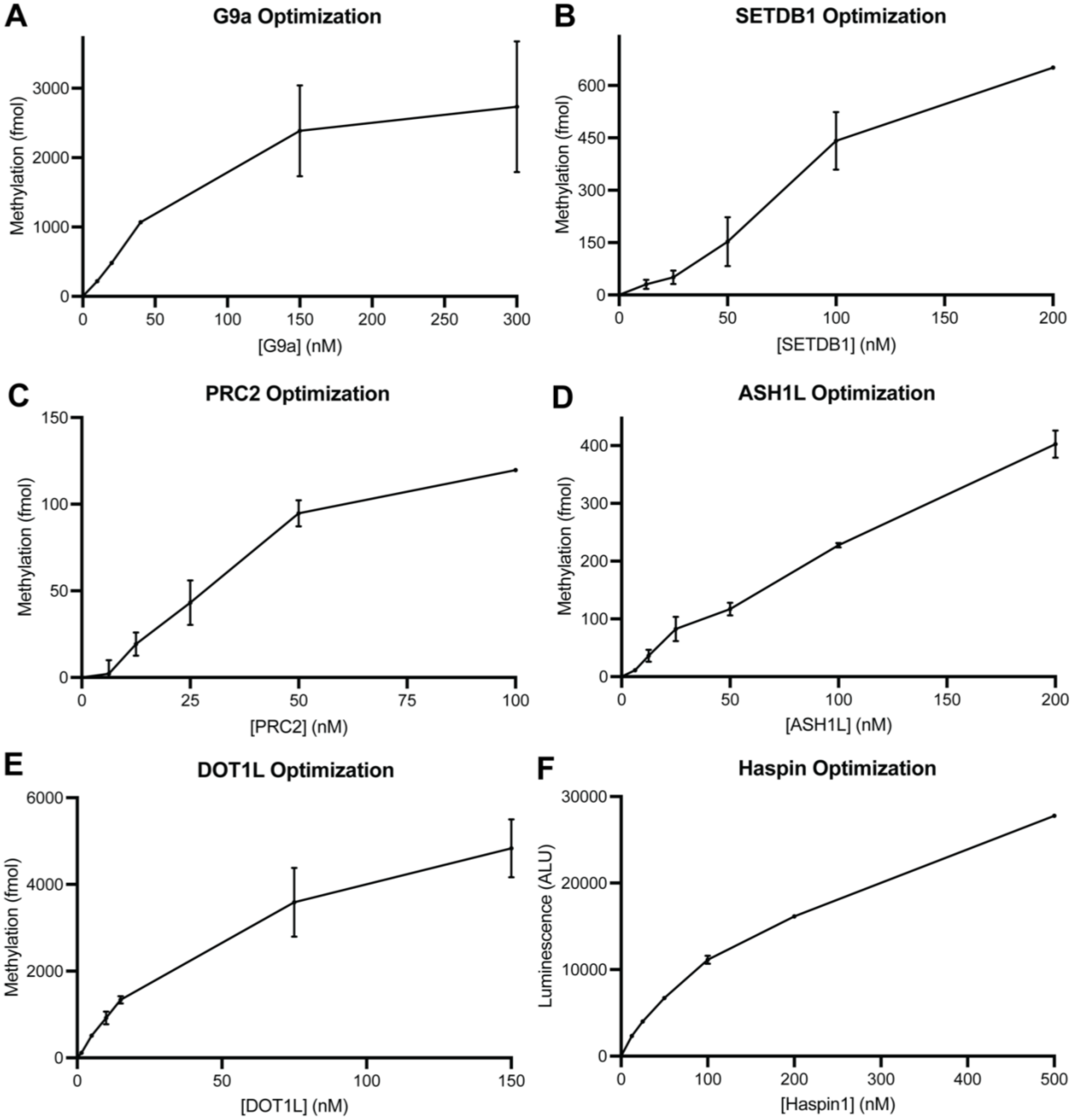

Supplementary Figure S4. Determination of optimal enzyme concentrations for *in vitro* methylation and kinase assays. Enzymes were titrated against 1 µg of chicken erythrocyte oligonucleosomes. Downstream assessment of either methylation or phosphorylation was performed as described (see Experimental Procedures). *n* = 2 for all assays shown. Error: SD.
